# Supporting culturally and linguistically diverse students during clinical placement: strategies from both sides of the table

**DOI:** 10.1186/s12909-015-0458-3

**Published:** 2015-10-15

**Authors:** Sharleen L. O’Reilly, Julia Milner

**Affiliations:** 1 School of Exercise and Nutrition Sciences, Deakin University, 221 Burwood Highway, Burwood, VIC 3125 Australia; 2 International Business School Suzhou, Xi’an Jiaotong-Liverpool University, 111 Ren Ai Road, Dushu Lake Higher Education Town, Suzhou Industrial Park, Suzhou, Jiangsu 215123 China

**Keywords:** Culturally and Linguistically Diverse (CALD) students, Placements, Challenges, Strategies

## Abstract

**Background:**

Increasing proportions of Culturally and Linguistically Diverse (CALD) students within health professional courses at universities creates challenges in delivering inclusive training and education. Clinical placements are a core component of most health care degrees as they allow for applied learning opportunities. A research gap has been identified in regard to understanding challenges and strategies for CALD students in health professional placements.

**Methods:**

A key stakeholder approach was used to examine barriers and enablers experienced by CALD students in clinical placement. Semi-structured focus groups with healthcare students (*n* = 13) and clinical placement supervisors (*n* = 12) were employed. The focus groups were analysed using open coding and thematic analysis.

**Results:**

Three main barrier areas were identified: placement planning and preparation; teaching, assessment and feedback; and cultural and language issues. Potential solutions included addressing placement planning and preparation barriers, appropriate student placement preparation, pre-placement identification of higher risk CALD students, and diversity training for supervisors. For the barrier of teaching, assessment & feedback, addressing strategies were to: adapt student caseloads, encourage regular casual supervisor-student conversations, develop supportive placement delivery modes and structures, set expectations early, model the constructive feedback process, use visual aids, and tailor the learning environment to individual student needs. The enablers for cultural & language issues were to: build language and practical approaches for communication, raise awareness of the healthcare system (how it interacts with healthcare professions and how patients access it), and initiate mentoring programs.

**Conclusions:**

The findings suggest that teaching and learning strategies should be student-centred, aiming to promote awareness of difference and its impacts then develop appropriate responses by both student and teacher. Universities and partnering agencies, such as clinical training providers, need to provide an inclusive learning environment for students from multiple cultural backgrounds.

## Background

An increasing proportion of Culturally and Linguistically Diverse (CALD) students are a feature of the growing diversity and multicultural societies in developed countries, of which Australia is no exception [1, 2]. Health professional courses will typically have higher than average CALD student numbers—usually a third of all students—compared with other university courses [3, 4]. These CALD students are at-risk of non-completion and lower academic performance [3, 5, 6]; some of the principle reasons being increased social isolation, engagement in paid work during term-time, stereotype threat and linguistic diversity [4–6]. For health professional courses the challenges become particularly evident in the clinical practice setting. Clinical educators are also confronted with providing adequate support to the CALD students through creation of equitable learning environments to enable student progress [7, 8].

Whilst the literature acknowledges the above barriers exist, mainly for medicine and nursing domains [4–6, 9], there is little evidence that encompasses the broader health professional disciplines [7, 10]. There is also a need to identify effective strategies to support CALD students engage in equitable clinical learning environments across a variety of settings [7, 10, 11]. Similarly, a gap between the experience of clinical educators and CALD students engaging with the proposed strategies endures. Clinical educators often describe university support in the area of cultural competency and awareness in the learning environment as insufficient [12]. Furthermore a disproportionate number of CALD students enrolled in health professional courses continue to make inadequate course progress [3, 13, 14]. This points to a disconnect between the problems identified and solutions generated, raising the question of whether they are authentic and reflective of the needs of the different stakeholders [13].

The catalyst for this research project was an internal report at a single institution finding CALD students on healthcare professional degrees averaged three additional placement weeks to achieve competency. This in addition to the identified gaps in the literature led us to explore the views of both CALD students and their clinical educators on the issues they saw as underpinning CALD students struggling on placement and potential solutions to those issues. Focus groups were selected to undertake the project and answer the following research questions:

1. What experiences and underlying issues do CALD students and their supervisors identify as likely to influence learning outcomes on placement?
2. What self-identified strategies and resources are likely to improve learning outcomes for CALD students on placement?

The goal of the project was to engage both main stakeholders (students and supervisors) in problem identification and resolution discussions, where themes can be identified and used to guide better placement experiences for all students.

## Methods

### Setting and participants

We used a focus group approach to explore the above research questions and had separate groups for healthcare students (who identified as CALD) and clinical placement supervisors. CALD students were recruited through advertisements within International student university newsletters and websites, posters in relevant Schools and brief student presentations. CALD students in their final year or a year with a large placement component within the university’s healthcare training courses (medicine, nursing, dietetics, occupational therapy and social work) were the recruitment focus. Clinical placement supervisors were recruited through an approach to heads of departments within services participating in the university’s placement programs to disseminate the supervisor’s focus group invitations.

### Data collection and analysis

Four CALD student and three supervisor semi-structured focus groups were held between September and October 2008. Student and supervisor focus groups were conducted separately due to concerns regarding the potential of existing or perceived power relationships to impact the ability of participants to engage in conversation. Approximately four students or supervisors participated in each individual focus group. The participant numbers reflect approximately 60 % dietetics supervisors/CALD students, 25 % social work CALD students and <5 % CALD nursing students/supervisors (based on internal enrolment and clinical placement data). The focus groups were conducted by a research fellow and research assistant under the guidance of the project’s multidisciplinary steering group composed of a variety of university staff involved in health professional training (occupational therapy, social work, nursing, medicine and dietetics) and international student welfare. The research assistant made notes during the focus group discussions, recording basic details such as demographic data, general flow of conversation and significant non-verbal communications. The focus groups averaged 60 min in duration. Basic ground rules and an introduction preceded the focus group sessions. Digital audio recordings of each session were transcribed professionally and were further annotated with coding to remove any identifiable information. Only the research team had access to personally identifiable information provided during the group sessions. Once the audio recordings were transcribed, verified and identifying data was deleted; the coded data were used for all analysis. Thematic analysis was the approach used for data analysis [15]; analysis began with independent open coding of the transcripts by the research fellow and research assistant, generating a list of content categories and sub-categories. These were reviewed, commented on and confirmed by members of the Project Steering Group. Emerging themes and linkages were used to develop an explanatory understanding of the findings. Ethics approval was granted from Deakin University Human Ethics committee in 2008, ethics number HEAG-H 84_08. Written informed consent was obtained from each participant prior to engaging in the focus groups.

## Results

### Participants

Thirteen students participated in the CALD student focus groups, 12 females and one male. The country of permanent residence profile for participants was 92 % Asian (Chinese, Chinese Malaysian, Chinese Singaporean, Japanese, Malaysian and Singaporean) and 8 % European (Norwegian). This is relatively reflective of figures given for overseas students in Australian health programs, 59 and 10 % respectively [16]. The mean student age was 29 (±5.6) years. Most students were in their 3rd year (*n* = 7) and 4th year being the next most common (*n* = 3), there were single student’s representing 1st and 2nd year plus one recent graduate.

Twelve supervisors participated in the clinical supervisor focus groups, all were female. The majority of supervisors (*n* = 10) were involved in dietetic supervision within their third and fourth years of training. The remaining two supervisors taught into the nursing course year’s one through three. Most supervisors identified with being between 30 and 40 years old (*n* = 8) with those in the 20–30 and 40–50 years groups having two supervisors each.

### Themes and sub-themes

Table 1 summarises the main challenges and the associated strategies identified by supervisors and students to increase CALD student success on placement. Illustrative quotes of the themes and sub-themes from the two participant groups are contained within Tables 2, 3 and 4.

**Table 1:** Summary of main themes resulting from CALD student and clinical placement supervisor focus groups

Theme 1		Theme 2		Theme 3	
Placement planning and preparation	Supportive strategies to improve outcomes	Teaching, assessment and feedback on placement	Supportive strategies to improve outcomes	Culture and language for placement	Supportive strategies to improve outcomes
Timely student preparation and orientation	Appropriate student placement preparation	Double-edged sword of regular assessment and feedback	Adapting student caseloads	Using English as a primary communication tool	Language building for communication
	Pre-placement identification of higher risk CALD students		Regular casual supervisor-student conversations		Practical communication approaches
			Supportive placement delivery modes and structures		
Supervisor training for CALD student supervision	Supervisory training focused on CALD students	Difference in approach to learning and teaching styles within placements	Early expectation setting and modelling of constructive feedback process	Greater Insight required into healthcare system, professional roles and broader social environment	Healthcare system awareness and how interacts with healthcare professions and how patients access it
			Visual aids and tailoring the learning environment		
			Placement delivery modes and structure		
Seeking more allocation of time for CALD students on placement				Social nature of Interpersonal relationships required on placement	Mentoring programs
				Pressures of self-care in a culturally foreign setting

**Table 2:** Verbatim statements for the theme “Placement planning and preparation”

Sub-theme	CALD Student challenges	Supervisor challenges	Supportive strategies
Supervisor training for CALD student supervision	*I’ve talked to one supervisor once…, and I said ‘What kind of training did you get from the Uni?’. And she said ‘Nothing… we didn’t get any training, the Uni just send us to here to supervise you guys’.* (Student group 2)	*I don’t think that they give probably enough support to their educators when you have a large group of students with different cultures”* (Supervisor group 2)	CALD student-focused supervisor training:
			*It would be nice maybe if the unis gave more time in teaching about international students to the facilitators, and even if they did a couple of workshops a year to the staff*…(Supervisor group 1)
			*I was thinking the Uni can give some …training to the clinical supervisor, before they supervise us…and* …[teach them about] *cultural things …, just let them know we have some language difficulties… I guess that would be great*. (Student group 2)
Timely student preparation and orientation	…[for] *first year students…*[there was] *a preparation for placement …, and they only talk about like ‘Be active in your placements… don’t be quiet’ and that’s it*. (Student Group 2)		Appropriate placement preparation:
			*I think it* [CALD placement preparation] *really has to… make sure that it actually runs throughout* [the course]… *and not… only on the third year when we’re going on placement then … realize the importance … I think it’s a bit too late in a way. (Student group 3)*
	*You do not know the expectation of… placement. Because* [in] *first year they had CALD* [placement preparation sessions]*, we went for it… … we did not see the relevance of it at the time* … (Student group 3)		Pre-placement identification of higher risk CALD students:
			*Early identification would be the key because if they do need extra time, extra anything, identifying earlier gives them more time, because I often find by the time they hit us they’ve only got their you know 6 weeks or whatever it is to reach competency and you can’t address everything.* (Supervisor group 1)
Seeking more allocation of time for CALD students on placement	*I find doing a placement… there’s issue with time management because of the workload. And there’s so much to do but… yet there’s so little time. So… I don’t know if I want more breaks, or… more time to do it*. (Student group 3)	*For me I try to treat them with the same expectations because they are going through the same course and potentially they are going to be applying for the same jobs, so trying to get them to the same standard. But having said that, you do realize when somebody’s struggling with the language or just generally cultural things, I guess we do try to make allowances by perhaps trying to dedicate a little more time than we might otherwise. But I typically expect the same from all students*…(Supervisor group 1)

**Table 3:** Verbatim statements for the theme “Teaching, assessment and feedback”

Sub-theme	CALD Student challenges	Supervisor challenges	Supportive strategies
Difference in approach to learning and teaching styles within placements	*…* [In] *our culture … a teacher tells us what to do… and this is right, rather than… the teacher asks you ‘So what do you think you should do? …* (Student group 3)	*Whether that’s, you know, sort of a learning style, sort of rote learning style, you know learning about a condition…you know learning about diabetes or a certain condition but they’re not really understanding that background to it and therefore being able to apply you know, it in different settings or different situations.* (Supervisor group 1)	Early expectation setting and modelling of constructive feedback process:
			*I took her in with my other student and then together we sort of did a like a peer… It enabled her to observe, but also to give constructive feedback to this other student and sort of see what, you know try and learn from that way.* (Supervisor group 3)
		*Putting the 2:1 supervision level has probably stressed the supervisors more when you’ve been given a CALD student and non-CALD, if that’s…But you know, when you’ve got two students and one is potentially a lot more demanding, I guess the 2:1 model actually puts even more stress on the supervisor* (Supervisor group 1)	Visual aids and tailored student learning environment:
			*… I had a good supervisor… and I was able to actually ask … a lot of things. And then she drew like pictures for me also,… she took time to explain,… really slowly to me … that was really good.* (Student group 3)
			*Understanding their learning style, like because they are quite challenged in the first place you kind of need to know a bit of detail about how they learn, and what they respond to earlier on rather than getting a week or so, because some of them, it takes a while to engage, so knowing that upfront might help in the early stages.* (Supervisor group 1)
Double-edged sword of regular assessment and feedback	*…sometimes I feel the clinical facilitator is more like an assessing officer rather than helping us. … I just felt that they always assess you with things, … test your knowledge…, rather than stay … and help you with other*… [things]. (Student group 2)	*They’re very polite—I don’t think like most students they don’t want to challenge a supervisor too much in case…there is repercussions, but they do repeatedly ask until you give feedback on a weekly basis that you want them to be more self directed and eventually it sinks in. You have to give specific examples when they ask this question you expect them to go back and do their own reading in their own time to confirm their knowledge*…(Supervisor group 1)	Regular casual supervisor-student conversations:
			*… I would like a bit more attention because when it comes to… some cultural issue, I would actually like to discuss a bit more with my supervisor… like, asking her certain things, but… because time is so limited on the ward…, she has to split her time between two students and… sometimes it’s just so hard to catch her… and really sit down and talk her, …that’s why I felt like… maybe a bit more time can be spent… talking to us in a way, like just chatting, so that we can bring up certain issues with her … and asking her questions, rather than… I feel that sometimes on the ward… there’s not much time for you to do things.* (Student group 3)
			Adapting student caseloads by supervisors:
			*I guess I’ve tried to sometimes screen patients so that I’m not setting up a student to go and fail I guess sometimes* (Supervisor group 3)
			Supportive placement delivery modes and structures:
			*Having the tutorials where you try and create an environment that was relaxed and open and they couldbring out their issues was really helpful I think, both for them and certainly for us as supervisors and educators. Just to try and gain a bit of an insight into where they’re coming from and what issues they had.* (Supervisor group 3) *…have larger blocks at one particular facility rather than chopping and changing every few weeks because that can be really disruptive and again blows the confidence and they feel they are starting back again.* (Supervisor group 1)

**Table 4:** Verbatim statements for the theme “Cultural and language issues”

Sub-theme	CALD Student challenges	Supervisor challenges	Supportive strategies
Using English as a primary communication tool	…[when] *the patient got different pronunciation. You know… it’s difficult for me to understand them sometime. So… if I can’t understand the patient… I can’t keep going* [with] *my assessment or… treatment. We don’t lack the knowledge, but… our language is a barrier.* (Student group 2)	*Because if they were better at that (writing) and better at being able to express themselves in writing I think they might find they do a bit better overall [on placement]. (Supervisor group 1)*	Practical placement communication tips:
			*Giving them a few key sentences that they can use and that they know are respectful and not too abrasive …(Supervisor group 1)*
			*The other thing is I’ll get a student to repeat what I have just told them, “Can you tell me”… So that I check the understanding. Because often they’ll smile and they’ll nod and they’ll say “Yes, yes”, “Do you understand that?” “Yes, yes”. “Can you explain to me what I’ve just explained to you if you had to go and explain that to a patient?” And that’s when they come unstuck because they often then haven’t really understood it.* (Supervisor group 2)
		*They are, possibly because of the language…possibly, they are a little less willing to…and it may be a cultural thing too—they may be less willing to ask questions, and less likely to admit that they don’t know something.* (Supervisor group 1)	
			Building language skills for everyday communication:
			*..in the third year…I…will go and find… a voluntary organization to work there… so that I start interacting… because the earlier you start… talking to them* [patients] *then… it becomes natural… after a while.* (Student group 3)
			*there could be some sort of structuring in the uni system to have, and maybe interesting for them too on a cultural level, maybe Australian culture evening or something once a month where they get together and talk about the footy or, you know, Australian, Australian slang, and make it sort of almost entertaining but useful. Useful stuff for them and try to engage Aussie kids to come as well so in the breaks they could have a bit of…bit of chatting, rapport building type conversation skills.* (Supervisor group 1)
Insight needed into the Australian healthcare system and society generally	[It]… *is good to know the basic structure [of the Australian health care system] but also… where you refer patients to after their discharge… they call it the ‘continuum of care’*…(Student group 2)	*Students who came from societies where it was a very patriarchal type of, you know, “I’m the expert and I tell you what to do and you do it”, and it just doesn’t work like that here. And they didn’t understand why we didn’t use that model, they just didn’t get it and even though we would tell them “You need to negotiate” and you need to motivate your patient and so on, they didn’t understand. I’m the person telling them what to do and they do it. It’s just such a different model.* (Supervisor group 3)	Healthcare system awareness and how it interacts with public and healthcare professions:
			*… it would have been helpful to have something about the Australian health care system… but we didn’t get that until… well into… the second semester of… final year… would have been helpful to at least know that… way earlier.* (Student group 2)
			*… watching Australian* [hospital] *drama… like, ‘All Saints’.* (Student group 3) [referring to providing an opportunity to understand slang in context of health care and people’s lives]
Social nature of interpersonal relationships required on placement		*And I suppose that probably really comes up in you know even that initial establishing a rapport with a patient and that initial ‘how you going, what have you been watching on TV’…you know we might talk about what’s on … or footy or whatever, but obviously that would be much more difficult and is much more difficult for those CALD students and you know, that is something we assess the students on is being able to put the patient at ease*…(Supervisor group 1)	Peer mentoring:
			*I think it would be good to have…, a mentoring system…* [using] *students above… especially if they are… international student… mentors… or… a small group… it doesn’t necessarily have to be one to one. You get can some… local students who are willing to mentor… then there can be some cross cultural learning as well.* (Student group 2)
		*They’ve had a very poor reception by staff and sometimes the facilitator there, like outright discrimination, doors being slammed in their face and told they’re not welcome if you’re not Aussie, which I just find is totally disgusting, disgraceful beyond comprehension. So they’re quite pleased some of them I think when they come to us and they realise that we’re not like that*. (Supervisor group 2)	
Pressures of self-care in a culturally foreign setting		*I was just going to say there’s also that extra element with a lot of CALD students in the lack of support they have at home as well, they’ve been away from their families for four years […]* (Supervisor group 3)	
		*[…] and they might have to catch a bus, a tram and two trains to get to placement in the morning. That is really stressful for them, and I think sometimes, yeah, like where they’ve been placed isn’t fantastic for them either. So I think that’s a big constraint as well, we’ve got students who are running late in the morning and they’re all flustered and it takes them half the morning to calm down and get into the frame of mind to get into placement. Yeah, I’ve found that to be a problem.* (Supervisor group 3)	

### Theme 1: placement planning and preparation

There were three sub-themes within this theme: provision of supervisor training around specific CALD student issues; undertaking student orientation and preparation in a timely manner; and CALD student placements requiring more time allocation on a regular supportive basis.

The workload of supervisors demands that they manage their own caseloads and provide high-level student support. However, the level of supervisory training appears to be inadequate when the topic of CALD supervision was discussed. Whilst training is provided by university staff, the overall level of supervisor awareness about the training was low with only one group mentioned it with a varying levels satisfaction on its relevancy to their needs. Other supervisors remarked on a lack of resources available to help them manage issues relating to CALD students. The lack of consistency in supervisor CALD training awareness and their training experience is a potential concern.

Student placement preparation is essential for the smooth transition from classroom to workplace. Whilst all courses have a placement preparation plan and orientation processes, there was an inconsistent level of experience reported. Within the student preparation process, it appears that the timing of learning activities within degree programs is critical—too early and it is undervalued, too late and the opportunity is missed.

Students as well as supervisors discussed the influence of time constraints on placement organisation, structure and duration, pace, rotations and capacity. Both groups wanted more time allocated to placement planning and the importance of time management on placement was repeatedly stated. Supervisors reported being conflicted on the notion of a ‘level playing field’ put forward by universities to ensure student equity during learning experiences. While they supported the notion in principle, they weighing up the needs of CALD students on placement and found it difficult to implement in practice. Supervisors also felt it was a university responsibility to ensure all CALD students were adequately prepared before going on placement.

The strategies proposed for this theme to improve outcomes were: appropriate student placement preparation; pre-placement identification of higher risk CALD students; and supervisory training focused on CALD students.

Preparation was universally held as being important and both groups stressed the need for it to be specific, focused and well timed within degree programs to increase its effectiveness and maximised student engagement. A wide variety of formats were proposed (lectures, tutorials, workshops, clinical lab session, role plays, practical applications) as well as integrating an extra stream of support tutorials solely for CALD students throughout a teaching semester. The conclusion was a need for more time, resources and specific support services to be put into placement preparation.

Early identification of at-risk CALD students by university coordinators was a commonly identified strategy by supervisors. In several degree programs formal peer-to-peer learning occurs on placement; supervisors suggested more feedback and information pre-placement on the suitability of potential student pairings to create better matches would increase student learning rather than detract from it.

The final strategy was the need for specific and skilled training to give appropriate support to CALD students. Supervisors noted the need for teaching time to be allocated in order for the training to occur and linking into existing “in-house” training about cultural awareness for common client populations would help reduce any overlap within placement organisations.

### Theme 2: teaching, assessment and feedback

The sub-themes were: the difference in approach to learning and teaching styles within placements, and double-edged sword of regular assessment and feedback.

CALD students and their supervisors reported experiencing a learning environment that the students may not be prepared for. Students may have well-developed rote learning strategies and the expectation of ‘learning on the run’ can be fundamentally challenging. Both participant groups commented on the impact of culture on learning/teaching styles with specific difficulties noted asking questions, giving peer feedback, undertaking self-directed learning and reflective practice. Differences in prior learning and teaching experiences, time constraints and language barriers seem to play a role and were mentioned on both sides. Certain styles of placement supervision appear to be more problematic for CALD students, specifically when they were in a two student to one supervisor ratio.

Students valued the opportunity to demonstrate competency once they acquired a skill and use regular feedback as a mechanism to improve their learning. Some CALD students reported feeling like they were constantly being assessed with little opportunity to practice skills before assessment and supervisory engagement in the process and providing feedback was varied. The process of formative assessment requires dialogue to occur between students and supervisors. Supervisors commonly reported low levels of CALD student engagement in the process yet at the same time they reported the same students regularly asked for feedback. More informal supervisor-student relationships presented other challenges where students saw supervisors more as friends leading to feedback not being accepted.

The strategies proposed for this theme to improve outcomes were: adapting student caseloads; regular casual supervisor-student conversations; supportive placement delivery modes and structures; early expectation setting and modelling of constructive feedback process; visual aids and tailoring the learning environment.

Supervisor judgement or ‘screening’ of patient types was put forward to ensure CALD students are not given overly complex cases too early in their placement. While this process is considered part of normal placement supervision, supervisors felt it was especially important for at-risk CALD students where complexity might not be simply their clinical condition but potentially those with complex social situations, low levels of English language skills and/or strong accents. Allocating time to have regular informal conversations and clarifying early on with CALD students that those conversations are not assessed was also proposed.

Creating a peer-support network and allocating supervisor time to engaging with the network to model good communication skills and increasing student confidence was identified by supervisors. Another strategy identified on several occasions was ensuring CALD students had longer placements blocks at a single location. This strategy helped to reduce student anxiety and the stress of adapting to different environments on a repeated basis.

Other practical suggestions around how supervisors support CALD students in their learning were clarifying expectations early on for tasks in different settings and modelling of constructive peer feedback conversation. Some supervisors took time to personalise teaching experiences—diagrams and illustrations were mentioned—for CALD students to meet their learning needs.

### Theme 3: cultural and language issues

The sub-themes were: interpersonal placement relationships; using English as a primary communication tool; insight needed into the healthcare system, professional roles and society generally; and self-care pressures in a culturally foreign setting.

Learning and working in a different cultural and linguistic setting presents a host of challenges. Both groups reported the social nature of healthcare work and the pressures that it creates for CALD students being an issue. Specific aspects identified were: student understanding and undertaking of professional interactions, differences in personal space interpretation, difficulty in being assertive or interacting in groups, CALD discrimination and display of emotions. The concept of personal space and the process of establishing rapport were culturally variable; both aspects were recounted as impacting patient interactions. Language issues and established local social networks affected interpersonal relationships between CALD students and their peers. One supervisor reported this behaviour as being such that it could be described as discriminatory within their workplace.

English language skills to gain entry into a university course does not equate directly to an ability to converse casually or communicate complex health messages in simple language. The more nuanced practice of communicating within a healthcare setting means that CALD students and their supervisors identified several aspects that need to be considered: gaining sufficient exposure and practice opportunities; adapting language to suit different contexts; supervisor bias of perceiving CALD student communication issues; and the extent of language skills impacting student assessment and progress. Just as language skill is crucial, understanding the complexity of the healthcare system is equally fundamental. Both groups discussed this aspect at length and how it will impact patient care, student functioning on placement and academic assessment. Supervisors noted that CALD students might lack social support to help with daily living issues and pastoral care. These issues extended to practical components of placement like transport to placement site resulting in exhaustion from the stress of relying on public transport to get to their final destination.

The strategies proposed for this theme to improve outcomes were: building language and practical approaches for communication; raising awareness of the healthcare system (how it interacts with healthcare professions and how patients access it); and mentoring programs.

CALD students put forward practical suggestions on addressing their issues communicating with peers, for example, doing voluntary work and being pro-active when engaging with non-CALD students. Supervisors favoured students having a more formal University-based integration of peer interactions, for example University workshop or events such Australian culture evenings. A practical communication strategy identified by both groups was getting students to ask their patients to speak a bit more slowly and to speak slowly to them to ensure the patient understood them. Another strategy mentioned by a student was to speak quietly when they were unable to hear the patient so the patient would raise their voice. Practical supervisor communication strategies included showing interest in the student’s background by asking questions and encouraging students to paraphrase/reflect back on what they understood the supervisor was explaining.

Pre-placement preparation was a key strategy to tackle CALD student awareness and understanding of the healthcare system; both groups identified it. Supervisor’s comments came from wanting to ensure time on placement was not wasted and needing students to be ready to learn during the valuable placement blocks. CALD students proposed any university preparation be very practical and include hands-on learning. Both supervisors and CALD students mentioned mentoring as a way of improving social interaction skills. CALD students supported a formal mentoring scheme where more senior CALD students supported more junior ones.

## Discussion

Our research found having a CALD background impacted on healthcare professional placements, as experienced by the students themselves and their clinical placement supervisors. There were three main themes identified as likely to influence learning outcomes—placement planning and preparation; teaching, assessment and feedback on placement; and cultural awareness and language skills for placement. The strategies identified to improve CALD student placement learning outcomes were: appropriately tailored training and preparation for both students and supervisors; early and regular interactions between supervisors and students including expectations setting and tailoring of the learning environment; language building and communication approaches as well as awareness of healthcare structures. Whilst strategies have been reported in the literature, these usually focus solely on the supervisor or the student. By including both stakeholders in our study and therefore identifying strategies by both students and supervisors, we see a picture of a broader learning environment that all students (CALD and non-CALD) as well as supervisors can benefit from.

Clinical placements are approached differently compared with other traditional learning environments, mainly because they are delivered by external organisations and universities have less control over the way resources—staff, timing and setting—are delivered. This issue creates a tension when trying to create an optimum learning environment and supports our finding that strategies need to be tailored to both the student learning needs but also around the needs of the learning environment, the clinical placement. One such approach to improving the learning environment is delivering diversity training, which is acknowledged as an important part of preparing all stakeholders involved for the placement setting within an Australian government report [17] and supported by other studies [7, 18]. However other research suggests the training be specifically focused on training teachers so they are equipped to work with students from different cultural backgrounds [19–21] and this was argued to be especially important if teachers have not had a ‘multicultural education’ [22]. Our findings support the need for training to be across the board (supervisors, CALD and non-CALD students), this being illustrated by supervisors finding non-CALD student diversity-related knowledge being quite variable and the execution of supervisory training to be patchy with mixed awareness of existing training opportunities. Putting university-paid incentives in place to motivate supervisors to attend training may be one way of improving standards within external organisations, mainly because other workload demands can easily be given priority within the busy healthcare setting [8].

The structure of the clinical learning environment needs to incorporate student support mechanisms that are equitable for all learners. We found support needed to include regular and early interactions—included setting expectations and tailoring of the learning environment—for positive placement outcomes. This approach is likely to be more time-intensive, with teachers having to invest more time for things like individual appointments for CALD students who identify early as finding the clinical setting challenging [23]. The learning environment also extends to language support—tailored to the clinical setting—which remains a critical factor for successful placement and academic outcomes. The literature supports this but is not specific about the best mode to provide it, both face-to-face and online delivery being reported as successful modes [24, 25].

University-based preparation is a typical component of the student learning experience, which our findings and others support as being useful in familiarising CALD students with clinical learning environment and healthcare system more generally [26]. In their systematic review on strategies for CALD students on clinical placements, Brennan et al. conclude that orientation programs for CALD student are beneficial if they were attended before the actual placement commences [8]. Our study supported those findings and identified an additional important caveat: if programs are offered too early within a course, students may fail to see their value and conversely, if offered too late within a course, students may have already experienced a common issue and feel unsupported. The sharing of more applied and practical communication tips was not an area directly identified in our findings, although many were provided when participants were prompted, and this could be an area where universities could facilitate this knowledge exchange. Creating university-led CALD student support groups has seen improved learning outcomes in the literature [20, 27], our findings suggest mentoring using more senior CALD students was a preferred strategy. What is more mentoring is important for all students to build their communication competency so it could be built in an equitable way into both university and clinical placement settings [28]. This draws us back to a central issue to this problem where CALD student issues are isolated a standalone problem rather than viewing them as part of the continuum of creating an effective learning environments for all parties involved.

### Strength and limitations

A major strength of this research is its stakeholder approach, rural and regional placement sites and a focus on solution generation from different stakeholder perspectives. However the student participants were predominantly from two healthcare professional backgrounds (nursing and dietetics) and a single institution; we do not know the extent of (or even if) these characteristics influence the perspectives reported. The dietetics and nursing supervisors had a broader perspective as they provided training for a range of universities and were from three separate clinical sites. Furthermore the experiences of male supervisors or CALD students may not be reflected within this cohort; the health professions represented in our focus groups are 90–95 % female predominated [29, 30] and as such, our participants were mostly female. The focus groups generated a large amount of data and saturation was reached for the themes identified but the degree to which they reflect the broader clinical supervisor and/or CALD student population can only be interpreted from the existing literature. University placement coordinators were not involved directly in this research, this was a conscious decision by the steering committee owing to their role being external to the clinical placement setting. This exclusion may be considered a limitation when examining the research area from a holistic learning design perspective.

## Conclusion

This study adds to the knowledge base on CALD students and their experience on clinical placements, by including the perspective of both supervisors and CALD students, and supports the need for the development of more inclusive strategies and clinical placements into the future. CALD students face significant challenges within the clinical placement setting, our study confirms these challenges need to be considered when developing clinical placement learning environments to ensure CALD students receive equitable and suitable support—the timing of which is critical to ensure they are effective. All learning environments need support from decision-makers within each setting and it is an important component that should be investigated in future work.

If the goal is to make structural changes to the learning environment of clinical placements, it needs to be ensured that decision-makers from both the University and clinical setting are in included in the process. Further research should include high quality evaluation of clinical learning environments and investigation of quality indicators that are feasible within a busy clinical setting to ensure that the process of delivering an equitable and inclusive learning can be monitored and used to guide improvement.

## Abbreviations

CALD: Culturally and Linguistically Diverse
